# Distribution pattern and prevalence of West Nile virus infection in Nigeria from 1950 to 2020: a systematic review

**DOI:** 10.4178/epih.e2020071

**Published:** 2020-11-26

**Authors:** Idris Nasir Abdullahi, Anthony Uchenna Emeribe, Peter Elisha Ghamba, Pius Omoruyi Omosigho, Zakariyya Muhammad Bello, Bamidele Soji Oderinde, Samuel Ayobami Fasogbon, Lawal Olayemi, Isa Muhammad Daneji, Muhammad Hamis Musa, Justin Onyebuchi Nwofe, Nkechi Blessing Onukegbe, Chukwudi Crescent Okume, Sanusi Musa, Abubakar Muhammad Gwarzo, Odunayo Oyetola Rahmat Ajagbe

**Affiliations:** 1 Department of Medical Laboratory Science, Faculty of Allied Health Sciences, Ahmadu Bello University, Zaria, Nigeria; 2 Department of Medical Laboratory Science, Faculty of Allied Medical Sciences, University of Calabar, Calabar, Nigeria; 3 WHO National Polio Laboratory, University of Maiduguri Teaching Hospital, Maiduguri, Nigeria; 4 Department of Medical Laboratory Science, Kwara State University, Malete, Nigeria; 5 Department of Medical Laboratory Science, Faculty of Allied Health Sciences, University of Maiduguri, Maiduguri, Nigeria; 6 Public Health In-vitro Diagnostic Control Laboratory, Medical Laboratory Science Council of Nigeria, Lagos, Nigeria; 7 Department of Medicine, National University of Samoa, Samoa; 8 Department of Medical Microbiology and Parasitology, Faculty of Clinical Sciences, Bayero University, Kano, Nigeria; 9 Department of Medical Laboratory Science, University of Nigeria, Nsukka, Nigeria; 10 Department of Strategic Information and Research, Institute of Human Virology, Abuja, Nigeria; 11 Department of Medical Laboratory Service, University of Nigeria Teaching Hospital, Enugu, Nigeria; 12 Department of Medical Microbiology and Parasitology, Federal University, Dutse, Nigeria; 13 Solina Center for International Development and Research, Abuja, Nigeria

**Keywords:** One Health, Zoonosis, West Nile virus, Nigeria, Pooled prevalence

## Abstract

**OBJECTIVES:**

West Nile virus (WNV) is a re-emerging mosquito-borne viral infection. This study investigated the pooled prevalence pattern and risk factors of WNV infection among humans and animals in Nigeria.

**METHODS:**

A systematic review was conducted of eligible studies published in PubMed, Scopus, Google Scholar, and Web of Science from January 1, 1950 to August 30, 2020. Peer-reviewed cross-sectional studies describing WNV infections in humans and animals were systematically reviewed. Heterogeneity was assessed using the Cochrane Q statistic.

**RESULTS:**

Eighteen out of 432 available search output were eligible and included for this study. Of which 13 and 5 were WNV studies on humans and animals, respectively. Although 61.5% of the human studies had a low risk of bias, they all had high heterogeneity. The South West geopolitical zone of Nigeria had the highest pooled prevalence of anti-WNV immunoglobulin M (IgM; 7.8% in humans). The pooled seroprevalence of anti-WNV IgM and immunoglobulin G (IgG) was 7.1% (95% confidence interval [CI], 5.9 to 8.3) and 76.5% (95% CI, 74.0 to 78.8), respectively. The WNV RNA prevalence was 1.9% (95% CI, 1.4 to 2.9), while 14.3% (95% CI, 12.9 to 15.8) had WNV-neutralizing antibodies. In animals, the pooled seroprevalence of anti-WNV IgM and IgG was 90.3% (95% CI, 84.3 to 94.6) and 3.5% (95% CI, 1.9 to 5.8), respectively, while 20.0% (95% CI, 12.9 to 21.4) had WNV-neutralizing antibodies. Age (odds ratio [OR], 3.73; 95% CI, 1.87 to 7.45; p<0.001) and level of education (no formal education: OR, 4.31; 95% CI, 1.08 to 17.2; p<0.05; primary: OR, 7.29; 95% CI, 1.80 to 29.6; p<0.01) were significant risk factors for WNV IgM seropositivity in humans.

**CONCLUSIONS:**

The findings of this study highlight the endemicity of WNV in animals and humans in Nigeria and underscore the need for the One Health prevention and control approach.

## INTRODUCTION

Globally, West Nile virus (WNV) is one of the most widely distributed arboviruses of public health significance in both humans and animals [1]. This mosquito-borne flavivirus is a member of the *Flaviviridae* family [2]. In nature, WNV is maintained in a zoonotic transmission cycle between birds and mosquitos, mainly *Culex* species. Susceptibility to WNV infection has also been indicated for many other vertebrate hosts, including reptiles, amphibians, and mammals [3]. Ecologically, horses and humans are “dead-end” hosts that do not play a role in the transmission cycle of WNV [3]. However, they can manifest severe disease or death as a consequence of WNV infection [3]. Approximately 80% of WNV infections in humans are inapparent, while in other persons, WNV produces flu-like malaise and severe neuroinvasive disorders that currently have no specific cure [4]. Fewer than 1% of WNV-infected persons develop severe WNV disease [4]. The severity of disease in this latter group of individuals is influenced by age, immune status, and terminal comorbidities [4]. The nonneurotropic form of WNV infection can either be mild or critical, with an incubation period of 3-14 days. It is usually accompanied by pyrexia, anorexia, body aches, swollen lymph nodes, and muscle and joint pains [4]. Although the pathogenesis of WNV infections has yet to be completely elucidated, certain studies have revealed the involvement of the host’s genetic factors as a predisposing factor for severe WNV disease [5,6].

Since the first discovery of the virus in 1937 in the West Nile district of Uganda [6], it has undergone significant geographical spread around the world through activities such as globalization, land use, and international travel. WNV infection was first identified in Nigeria in the 1950s [4,7]. Since then, infection with the virus has been reported in many parts of Nigeria and subsequently in several other countries across the sub-Saharan African region [7].

Nigeria is a nation in West Africa situated at a latitude of 9.0820°N and a longitude of 8.6753°E. It shares land borders with the Republic of Benin in the west, Chad and Cameroon in the east, and Niger in the north-east and north-west zones of Nigeria. The southern part of Nigeria is characterized by a tropical rainstorm climate, which is affected by storms that originate from the south Atlantic Ocean and move towards the southwest of Nigeria. Its warmth and high humidity give it a substantial propensity to rise and create abundant rainfall [8].

**Figure 1.**
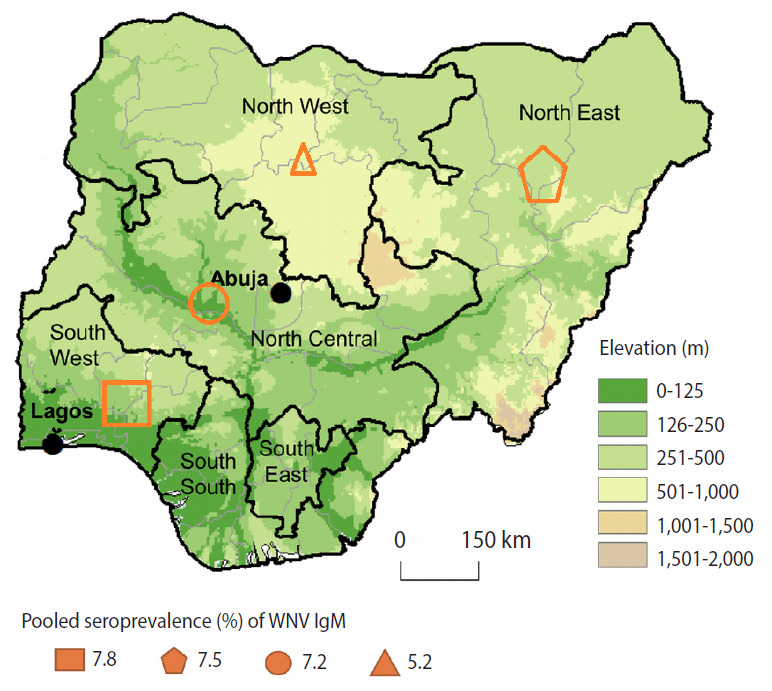
Geopolitical zones of Nigeria, their corresponding elevations above sea level, and pooled West Nile virus (WNV) immunoglobulin M (IgM) seroprevalence^1^. WNV data were not available in the South East and South South zones. ^1^Seroprevalence: percentage of individuals with antibodies to WNV in a study population over the specific period.

The tropical monsoon climate has temperature ranges that are consistent throughout the year [8]. The southern part of the country encounters overwhelming and abundant rainfall [8]. The total annual rainfall received in this region is high, above the 2,000 mm rainfall threshold that defines the tropical rainforest climate, which is characterized by significant forest cover. However, more than 4,000 mm of rain can be observed in the South South geopolitical zone of Nigeria (Figure 1).

The Sahel climate is predominates in the northern part of Nigeria, with a lower total annual rainfall than in the southern and central parts of Nigeria [8]. The rainy season in the northern part of Nigeria usually lasts from June to September (3 to 4 months). The rest of the year is hot and dry, with temperatures climbing as high as 40°C [8]. The Sahel climate predominantly provides the savannah vegetation, with very little tree cover, extensive grasses, and significant amounts of dust from the Sahara Desert [9].

As of November 4, 2020, Nigeria has a population of > 207 million inhabitants and a population density of 226 per km^2^ [9], making it the most populated African country. Nigeria has been reported to have endemic arboviral, malarial, and typhoid infections [10]. Due to the similarity of the febrile illnesses caused by these infections, almost all fever cases are ascribed to malaria or typhoid unless otherwise confirmed through accurate laboratory investigations [10-12]. Hence, prevention and control efforts for WNV should substantially rely on effective surveillance of the infection in birds, vectors, animals, and humans. Although several studies have explored different aspects of WNV epidemiology in some geopolitical zones of Nigeria, there remains a paucity of data about its driving factors. Thus, understanding the epidemiology of WNV in Nigeria faces several challenges, including an inadequate knowledge of the nature of WNV disease among healthcare professionals, underdiagnosis (and/or misdiagnosis), poor diagnostic infrastructure for arboviruses, and a lack of comprehensive and consistent surveillance systems. These have resulted in a gap in available data regarding the distribution pattern and prevalence of WNV infection in Nigeria. Hence, this systematic review was conducted to provide a comprehensive overview of the epidemiology of WNV among human and animal populations in Nigeria through a systematic review and meta-analysis of original studies conducted and published from January 1, 1950 to August 30, 2020. The findings of this study will help to build effective intervention measures to curb the burden of WNV infections in humans and animals.

## MATERIALS AND METHODS

### Data sources and search strategy

Relevant articles were searched, screened, and included in this study according to the PRISMA (Preferred Reporting Items for Systematic reviews and Meta-Analyses) criteria [13]. Articles were searched through the Web of Science, Scopus, PubMed, Google Scholar, and Index Medicus as an African database using different combinations of the following keywords: “West Nile virus,” “prevalence,” “serological detection,” “West Nile fever,” “WNV,” and the names of Nigerian cities and states. All selected databases were searched for only English-language full-text original articles published from January 1, 1950 to August 30, 2020. Multiple sources for the article search were used to enhance the sensitivity of finding relevant articles.

### Review selection

The studies identified through electronic and manual searches were listed in EndNote X9 (Clarivate, London, UK). After the exclusion of duplicate citations, all authors separately reviewed the titles and abstracts of selected articles. The relevant full-text studies obtained were assessed for eligibility and risk of bias. All original articles from peer-reviewed scientific journals with a crosssectional or survey design that estimated the prevalence of WNV infection in humans and animals were considered potentially eligible for inclusion in this review. Relevant studies whose full-text was not available were sought by contacting the corresponding author through email. All studies outside of Nigeria were excluded. Any disagreements in data interpretation between the authors were resolved through collective discussion.

### Literature search strategy

For the purpose of this study, we utilized the PICOS questions as a framework related to reviews of intervention effectiveness. In the PICOS focused search strategy structure, (P) refers to the population or patients, (I) to intervention or exposure, (C) to the comparison, (O) to the outcome, and (S) to the study design. We used WNV infection as P; anti-WNV immunoglobulin M (IgM), anti-WNV immunoglobulin G (IgG), WNV-neutralizing antibodies, or WNV RNA positivity as I; no WNV infection, WNV seronegativity, or WNV RNA negativity as C; fever, febrile illness, and WNV disease as O; and a cross-sectional study design as S.

### Assessment of study bias

The risk of bias in primary studies was assessed using the Cochrane method. The sample size of each study was included as a criterion for determining the risk of bias, as described by Humphrey et al. [14]. WNV prevalence studies were evaluated in the following 3 domains: sampling technique, participation level of the subjects, and WNV test method. Studies were categorized as having a low risk of bias if they used random sampling techniques, > 80% participation by respondents, and the use of either a virus neutralization test or a nucleic acid amplification test to determine WNV prevalence in the overall study population [15,16].

Studies that did not provide information for the 3 aforementioned domains were classified as having an unclear risk of bias. When a study did not satisfy 1 of the domains, it was considered as having a moderate risk of bias. If information was not available on 2 domains, the study was classified as having a high-risk of bias. The use of a random sampling technique was only applicable for studies on the general population because there would be significantly high bias in selecting participants with acute fever infections from healthcare facilities. Studies on humans were considered to have high precision if their sample sizes were greater than 100 [17].

### Data extraction and curation

Appropriate data were extracted from the selected studies into an Excel spreadsheet. Data were extracted based on the first author’s name, year of publication, state/city, sample size, participants’ age, sex, and other available socio-demographic variables (for human studies), animal species (for animals’ studies), and WNV prevalence by assay type. Data cleaning was done to identify and correct errors during collection to minimize their effects on the results.

### Statistical analysis

The crude prevalence of WNV infection was first calculated based on the crude numerators and denominators provided by all eligible studies. The heterogeneity of the pooled prevalence (PP) was calculated using the chi-square test on the Cochrane Q statistic, which was quantified by the I^2^ value, assuming that I^2^ values of 25%, 50%, and 75% represented low, medium, and high heterogeneity, respectively. MedCalc version 2019.19.0.7 (MedCalc Software, Ostend, Belgium) was used for all statistical analyses. The p-values less than 0.05 were considered to indicate statistical significance, and 95% confidence intervals (CIs) were calculated.

### Ethics statement

As the present study was a systematic review, no ethics statement was needed.

## RESULTS

### Search output

**Figure 2.**
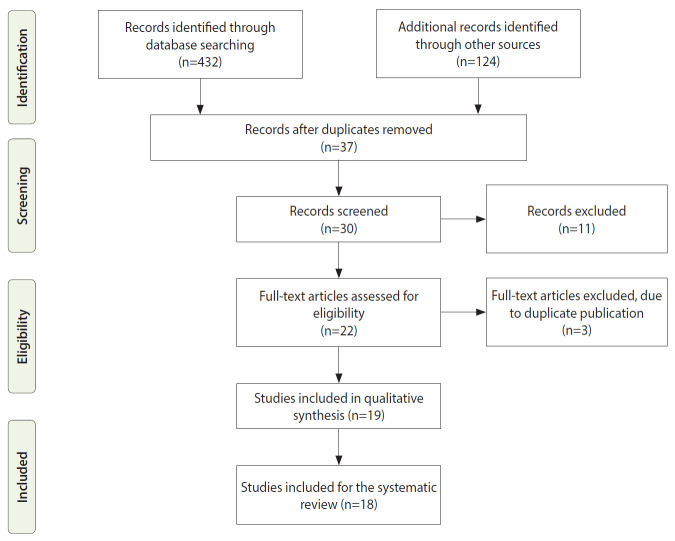
PRISMA (Preferred Reporting Items for Systematic reviews and Meta-Analyses) flow diagram of the search strategy for the inclusion of published studies.

We identified 432 records; after the elimination of duplicates, 37 remained. After screening titles and abstracts, we assessed 30 full-text articles for eligibility. Finally, 18 full texts were included because they were the only available cross-sectional studies (Figure 2). Upon further screening, it was determined that 7 studies identified WNV infection using anti-WNV IgM enzyme-linked immunosorbent assay (ELISA) in humans, 3 used anti-WNV IgG ELISA, 3 used WNV RNA reverse-transcription polymerase chain reaction (RT-PCR), 2 used a plaque reduction neutralization test (PRNT), 1 used a complement fixation test, 1 used hemagglutination inhibition, and 1 used a mouse protection test (Table 1). Seven of the studies on WNV in humans (53.8%) were conducted in the South West zone of Nigeria, 4 (30.8%) in the North East zone, and 1 (7.6%) each from the North West and North Central zones. No WNV studies were reported from the South South or South East zones of Nigeria (Table 1).

### West Nile virus immunoglobulin M seroprevalence among humans in Nigeria

A total of 13 human prevalence studies for WNV were identified, of which 12 estimated seroprevalence in febrile patients and 1 in apparently healthy blood donors. The studies covered 6 of the 22 states of Nigeria and were published from 1959 to 2020. The majority (61.5%) of the human studies had a low-risk of bias. All of the studies had high heterogeneity.

The South West zone of Nigeria had the highest PP of anti-WNV IgM (7.8%), followed by the North Central (7.5%), North East (7.2%), and North West (5.2%) zones. However, no records were reported from the South East and South South zones (Figure 1). There was no significant association of anti-WNV IgM PP with the geopolitical zones in Nigeria (p=0.508).

In humans, the pooled WNV IgM and IgG seroprevalence rates were 7.1% (95% CI, 5.9 to 8.3) and 76.5% (95% CI, 74.0 to 78.8), respectively. Out of the 1,473 pooled subjects tested, the WNV RNA prevalence was 1.9% (95% CI, 1.4 to 2.9). However, out of 2,164 pooled subjects, 14.3% (95% CI, 12.9 to 15.8) had WNV-neutralizing antibodies. Of note, a study reported a 47.0% WNV non-structural protein 1 (NS1) prevalence in febrile persons (Table 2).

### West Nile virus seroprevalence among animals in Nigeria

A total of 5 animal WNV prevalence studies were identified, of which 2 were from the North East zone, 1 was from the South West zone, and the remaining studies collected animals from the North East and South West zones (Table 3). In animals, the pooled WNV IgM and IgG seroprevalence were 90.3% (95% CI, 84.3 to 94.6) and 3.5% (95% CI, 1.9 to 5.8), respectively. Out of 620 pooled animals, 20.0% (95% CI, 12.9 to 21.4) had WNV-neutralizing antibodies (Table 4). Of note, a study reported 3.5% WNV IgG prevalence in pigeons (Table 3).

### Pooled risk factors of West Nile virus immunoglobulin M seropositivity in Nigeria

The pooled seroprevalence of WNV IgM was significantly higher among subjects younger than 17 years of age (22.9%) than among those over 17 years of age (7.4%; p<0.001). The pooled seroprevalence of WNV was somewhat higher in males (10.4%) than in females (8.0%), and among suburban residents (17.0%) than among those who resided in urban settlements (7.6%). The pooled seroprevalence of WNV was highest among subjects with primary education (15.2%), followed by among those without a formal education (9.6%), those with a secondary education (5.9%), and in those with tertiary education (2.4%); this difference was statistically significant (p<0.05) (Table 5).

Subjects who were unmarried (single) had a somewhat higher pooled WNV IgM seroprevalence (8.9%) than those who were married (4.8%). Persons who resided in areas with rainforest vegetation had the highest pooled WNV IgM seroprevalence (7.8%), followed by those in the Sahel (7.2%) and those in regions with savannah vegetation (6.6%). Subjects who lived in locations with a predominantly low environmental temperature (25°C) had a slightly higher pooled WNV IgM seroprevalence (7.7%) than those who resided in areas with a predominantly high temperature (38°C) (6.9%).

Those who lived in areas with 6 or more months of annual rainfall had a somewhat higher pooled WNV IgM seroprevalence (7.7%) than those who resided in areas with less than 6 months of annual rainfall, 6.9%. Those who did not consistently use mosquito nets had a slightly higher pooled WN IgM seroprevalence (6.8%) than those who did (6.1%). Bivariate logistic regression showed that age (odds ratio [OR], 3.73; 95% CI, 1.87 to 7.45) and level of education (OR, 7.29; 95% CI, 1.80 to 29.6) were significant risk factors for WNV IgM seropositivity (p<0.05) (Table 5).

## DISCUSSION

The mosquito-borne WNV is endemic in a vast geographical area, including Nigeria. However, the distribution pattern and prevalence of WNV in the 6 geopolitical zones of Nigeria have been poorly studied. To address this gap, this study was conducted as a systematic review of WNV prevalence studies conducted among humans and animals in Nigeria.

Seroprevalence and molecular studies of WNV have been done in 6 of the 36 states of Nigeria and Abuja (the federal capital of Nigeria). Although the presence of WNV infection remains unknown in locations with no available data (n=30), it can be inferred that the virus probably circulates within these locations as well, particularly considering the rapid interstate travel and similar climatic and environmental factors that favor the transmission of WNV. These observations may support a hypothesis according to which WNV has dispersed across states, affecting localities adjacent to infected areas [35]. The argument is further strengthened if we consider the transmission route of WNV, which is similar to that of other mosquito-borne viruses such as dengue and yellow fever [36]. The interstate spread of WNV may be quite easy and rapid, since it can be transmitted through a broad range of vectors and reservoirs [37].

In Nigeria, WNV antibodies were first reported in 1959 [7]. Subsequently, a few studies have detected serological markers of WNV in the general population in some cities of Nigeria, especially Ibadan and Maiduguri. In our meta-analysis, the overall pooled WNV IgM and IgG seroprevalence was 7.1% and 76.5% in humans, respectively; these values are higher than those reported in a systematic review of the prevalence of WNV in the Middle East and Mediterranean countries [37].

The majority of the serological surveys included in our study used enzyme immunoassay (EIA) protocols to detect WNV antibodies. Even though this protocol is easy to use, sensitive, and readily available, its epidemiological application is limited by the potential for cross-reaction with antibodies of other flaviviruses, due to similar nucleotide homology they share and/or influence of vaccination-induced antibodies for yellow fever virus [12,38]. Consequently, the use of EIA protocols for serodiagnosis of WNV in people with a history of vaccination against any related flaviviruses can yield false-positive results [39]. To confirm an EIA seropositive result, serum samples need to be subjected to PRNT, a gold standard for WNV serodiagnosis [10]. However, the major limitation of PRNT is its low sensitivity for serological studies, as it can only detect antibodies at levels that can neutralize WNV; therefore, its use is limited in weakly-exposed people [39]. Despite these limitations, the 14.3% PP for neutralizing antibodies to WNV is relatively high and indicative of endemicity in Nigeria.

In this systematic review, almost all persons with either serological or genomic evidence of WNV infection demonstrated undifferentiated symptoms (mainly fever and headache) suggestive of WNV infection. Indeed, these symptoms are also nonspecific to WNV. Hence, WNV-infected individuals could be misdiagnosed with other febrile illnesses, especially in areas with evidence of flavivirus co-circulation [40]. Consequently, WNV testing using RT-PCR should be considered as part of the differential diagnosis for patients presenting with non-specific febrile illnesses. While RT-PCR is considered very accurate, paired WNV IgM could also be performed. The combination of WNV IgM and viral RNA can further enhance the detection rate of WNV [40,41]. However, among the 13 studies on human populations included this review, only 3 used a combination of serological and molecular assays for the diagnosis of WNV infection [10,22,25].

Since some cross-reactivity has been observed between WNV IgG antibodies with antibodies to other flaviviruses, analyses of the serological detection of WNV need to be consolidated, preferably using antigen-based EIAs such as the recombinant NS1 antigens. To this end, a serosurvey for the detection of the seroprevalence of WNV in Maiduguri in the North East zone of Nigeria reported a rate of 85% among febrile persons [10]. These impressive data confirm the circulation of WNV in the area, as reported previously by Baba et al. [21], who presented data on a large number of individuals exposed to WNV infection. This assay has been used to increase the specificity of WNV serodiagnosis. It has been useful for differentiating flavivirus infections by targeting specific epitopes during the acute phase of WNV infection [42].

The analysis of risk factors for WNV prevalence showed no association between WNV IgM seropositivity and the available socio-demographic variables of participants in the pooled studies. However, we found an association of WNV prevalence with the age and level of education of the pooled participants. Of note, the pooled seroprevalence of WNV IgM in this study was significantly higher among those ≤ 17 years of age than among those > 17 years of age. This suggests relative protection in older subjects. The decrease in IgM with age from childhood suggests that primary infection occurs early in life and provides protection in adulthood. Furthermore, it is possible that those < 17 years of age were less occupationally active, stayed indoors more than adults, and were expected to participate more in domestic activities. This might have increased their risk of being bitten by the daytime-biting mosquito vectors of WNV. These mosquitoes readily thrive in water collection sites. Similar findings were reported in a West African study [43].

Even though the distribution of *Culex* spp. is ubiquitous in subSaharan Africa, its overall population density tends to be higher in areas in proximity to the thick rain forest [11]. This may explain why the majority of available studies were from the South West zone and neighboring cities. Furthermore, temperature, humidity, and enzootic interactions, which are different across geopolitical zones, may also influence the density of WNV vectors and infections. Anti-WNV IgM was predominantly observed among those with no formal or only primary education. In addition, a higher seroprevalence of WNV infection was found among teenagers and children (≤ 17 years). The level of education variable represents participants’ education status at the time of participation. Thus, it appeared that those with a lower education status (primary or no education) had a higher pooled seroprevalence of antiWNV IgM (i.e., were more likely to be infected) than those with secondary and tertiary education. This finding is consistent with the report of Ma‟aji [18]. This may be because literacy and public education generally represent improved socioeconomic status, which leads to increased chances of access to medical care and therefore a reduced likelihood of exposure to the disease.

Although no significant association was found between the PP of WNV and the geopolitical zones of Nigeria, the South West and North East zones had the highest pooled seroprevalence of pooled WNV IgM. However, no records were reported from the South East and South South zones. This could be due to the presence of World Health Organization virology laboratories, which have permitted active arbovirus studies in these zones. Hence, the absence of WNV records in other zones could reflect the misdiagnosis of WNV [21]. In addition, this report aligns with reported associations of a high prevalence of WNV infection in communities proximal to rainforest vegetation in the South West zone of Nigeria, or where large-scale tree planting is conducted to fight desertification and erosion in the North East zone of Nigeria. These factors provide ideal breeding conditions for WNV vectors [25].

For the purposes of this study, we screened and included 5 eligible serological studies on WNV infections in animals in Nigeria. These studies were conducted in 4 cities in 3 states of Nigeria. All of these studies evaluated serological evidence of WNV infection among domestic animals. Since 1990, the highest prevalence of WNV among domestic animals has been reported among horses in the cities of Ibadan and Maiduguri [7]. The high pooled seroprevalence of 20.0% of neutralizing antibodies in animals and variation across the geopolitical zones of Nigeria indicates favorable conditions for the circulation of WNV in these animals. In locations with recorded animal WNV seropositivity, it is imperative to implement robust WNV infection prevention and control measures to reduce the risk of zoonotic transmission.

A study conducted in the city of Bauchi (in the North East zone of Nigeria) reported an approximately 3.5% prevalence of WNV IgG in pigeons. A comparable report reported levels of WNV IgG in pigeons that migrate to urban centers in search of food and nesting areas [44]. These pigeons come into direct proximity with humans, enhancing the zoonotic transmission of WNV from the birds to humans. Subsequent studies have demonstrated pigeons as a potential vertebrate reservoir host of WNV [44,45]. To further demonstrate the capacity for the transmission of WNV in birds, a Eurasian colored dove experimentally infected with WNV infection was discovered to have sufficient WNV viremic levels, which were sufficient for transmission to other animals and humans [46]. Although WNV is technically not transmitted to humans from birds, the WNV infections in pigeons reported in the current systematic review were from the urban areas of Bauchi in the North East zone of Nigeria, where they are domesticated in close proximity to humans. Hence, this city may have ongoing WNV transmission, which presents a potential danger to people who might be bitten by infected mosquitoes that come into contact with WNV-infected birds [47]. Based on this evidence, mosquitoes and birds may been considered to play a significant role in the life cycle of WNV.

Despite the critical role and abundance of WNV mosquito vectors in Nigeria [11,47], only 1 study investigated the presence of WNV in mosquito vectors in Nigeria [47], and no WNV was detected. However, *Cx. pipiens* and *Cx. quinquefaciatus*, the primary vectors of WNV, were reported to be present in the cities of Abeokuta, Lagos, and Ibadan in the South West zone of Nigeria [11]. However, the detection or WNV in *Culex* spp. alone does not confirm mosquitos as a competent vector for WNV. Aside from *Culex* spp., WNV has also been reported in Aedes and Mansonia mosquitoes [37].

The major limitation of this systematic review is the unavailability of comprehensive data. First, there is a scarcity of seroprevalence studies of WNV in Nigeria, and the standard of documented information varied. For instance, several accessible studies of humans focused mainly on adults. Most studies did not report the prevalence of WNV infection according to other socio-demographic variables. Accordingly, the distribution of WNV according to age and sex remains obscure in Nigeria and needs further investigation with unbiased samples. Although this meta-analysis provided a reasonable basis for comprehensive data about the presence of acute and previous exposure to WNV infection in most included studies, the data can hardly be utilized to accurately determine the true prevalence and state of WNV spread in Nigeria. Hence, there is a need for standardized seroprevalence studies at the national level to appraise the epidemic status and potential for future WNV outbreaks. Finally, there was substantial within-country heterogeneity in the prevalence of WNV infection. This might be due to diversity in the geopolitical zones, study populations, and the different sample sizes of the studies. A common source of bias in WNV prevalence studies relates to whether the sample is drawn from a hospital or the community. This could also be a potential limitation of this study.

Most of the included studies dealt primarily with the seroprevalence of WNV antibodies, while very few studies reported molecular prevalence. This is also a limitation of this review. To address this gap, there will be a need for a multi-disciplinary approach with scientists and researchers from medical laboratory science, human and veterinary medicine, and other disciplines in the life and environmental sciences.

## CONCLUSION

This first systematic national assessment of WNV prevalence provides evidence confirming the transmission of WNV in Nigeria, as almost all studies showed evidence of WNV infection in humans and animals. The findings from this study highlight the endemicity of WNV infection in animals and humans in Nigeria and underscore the need for the One Health strategic approach to the prevention and control of WNV infection and vectors. Finally, there is a need to promote and build more research capacity in the future to obtain more data on the phylogenetics of WNV in both humans and animals for drug and vaccine design/development to aid containment and case management.

This study provided information on WNV-related seroprevalence and molecular-confirmed WNV infections. However, there are still no national WNV data in Nigeria, as the country does not have active surveillance for WNV infections. It is imperative to consider consistent surveillance of WNV infection and prompt management of identified WNV disease in clinical practice. In most countries, WNV is legally designated as an infectious disease that must be reported to the Ministry of Health.

## Figures and Tables

**Table 1.** Characteristics of human studies included in this systematic review

Characteristics	Frequency
Year of publication search (range)	1950-2020
Period of inclusion of participants	1959-2020
Age range (yr)	1-80
Sex, n (%)	
Male	740 (52.8)
Female	748 (42.2)
Study area	
Urban	4
Suburbs	9
Both	0
Geopolitical zone	
North East	4
North West	1
North Central	1
South West	7
South East	0
South South	0
IgM ELISA	7
IgG ELISA	3
RNA RT-PCR	4
PRNT	2
HI	1
CFT	1
Mouse protection test	1
Clinical presentation	
Acute febrile illness	12
Apparently healthy	0
Blood donors	1

Ig, immunoglobulin; ELISA, enzyme-linked immunosorbent assay; RT-PCR, reverse-transcription polymerase chain reaction; PRNT, plaque reduction neutralization test; HI, hemagglutination inhibition; CFT, complement fixation test.

**Table 2.** Prevalence studies on human West Nile virus infections in Nigeria (n=13)

Study	State/city	Sample size, n	Prevalencen, %					Bias assessment
			IgM ELISA	IgG ELISA	Neutralizing antibody serology	RT-PCR	NS1 ELISA	
Oderinde et al., 2020 [10]	Maiduguri	200	36.0	85.0	-	6.5	47.0	Low
Ma‟aji, 2017 [18]	Zaria	135	5.2	-	-	2.9	-	Low
Macnamara et al., 1959 [19]	Ibadan	207	-	-	MPT: 9.7	-	-	Low
Olaleye et al., 1990 [20]	Ibadan	304	-	-	HI: 40.0	-	-	Low
Baba et al., 2013 [21]	Maiduguri	310	-	-	PRNT: 25.0	-	-	Low
Adesina et al., 2017 [22]	Ile-ife	165	-	-	-	3.6	-	Low
Bukbuk et al., 2017 [23]	Maiduguri	200	-	-	MNT: 9.5	-	-	Unclear
Oladipo et al., 2018 [24]	Ogbomoso	93	18.3	-	-	-	-	Moderate
Baba et al., 2006 [25]	Maiduguri	973	1.2	80.2	-	0.6	-	-
Opaleye et al., 2014 [26]	Osun State	185	0.0	-	-	-	-	High
Omilabu et al., 1990 [27]	Ibadan	170	-	-	CFT: 65.0	-	-	Moderate
	Ogbomoso							
Kolawole et al., 2015 [28]	Ogbomoso	93	12.9	19.4	-	-	-	-
Kolawole et al., 2018 [29]	Ilorin	200	7.5	-	-	-	-	Low

Ig, immunoglobulin; RT-PCR, reverse-transcription polymerase chain reaction; NS1, non-structural protein 1; ELISA, enzyme-linked immunosorbent assay; MPT, mouse protection test; HI, hemagglutination inhibition; PRNT, plaque reduction neutralization test; MNT, microneutralization test; CFT, complement fixation test.

**Table 3.** Summary of prevalence studies on West Nile virus in animals in Nigeria (n=5)

Study	State/city	Species	Sample size, n	Prevalence, %			Bias assessment
				IgM ELISA	IgG ELISA	Neutralizing antibody serology	
Baba et al., 2014 [30]	Maiduguri	Camel, donkey, horse	250	-	-	PRNT: 13.2	Low
Sule et al., 2016 [31]	Ibadan	Horse	145	90.3	-	-	High
	Ikoyi						
	Ajah						
Olaleye et al., 1990 [32]	Maiduguri	Camel, goat, cattle, sheep	200	-	-	HI: 17.5	Moderate
	Ibadan						
Waziri et al., 2018 [33]	Bauchi	Pigeon	376	-	3.5	-	Moderate
Omilabu et al., 1990 [34]	Maiduguri	Camel, cattle, goat	170	-	-	CFT: 33.0	Low
	Ibadan						

Ig, immunoglobulin; ELISA, enzyme-linked immunosorbent assay; PRNT, plaque reduction neutralization test; HI, hemagglutination inhibition; CFT, complement fixation test.

**Table 4.** PP of WNV among humans and animals in Nigeria

WNV marker	No. of studies	No. of subjects	PP (95% CI)	H	I^2^	p-value
Humans						
IgM seroprevalence	7	1,879	7.1 (5.9, 8.3)	292.3	92.4	<0.001^1^
IgG seroprevalence	3	1,266	76.5 (74.0, 78.8)	184.1	88.5	<0.001^1^
Neutralizing antibody prevalence	6	2,164	14.3 (12.9, 15.8)	548.6	98.5	<0.001^1^
Viral RNA prevalence	4	1,473	1.9 (1.4, 2.9)	33.3	25.6	
Animals						
IgM seroprevalence	1	145	90.3 (84.3, 94.6)	NA	NA	NA
IgG seroprevalence	1	376	3.5 (1.9, 5.8)	NA	NA	NA
Viral RNA prevalence	NA	NA	NA	NA	NA	NA
Neutralizing antibody prevalence	3	620	20.0 (12.9, 21.4)	25.8	48.5	0.072

PP, pooled prevalence; WNV, West Nile virus; CI, confidence interval; H, Cochrane Q, chi square for heterogeneity; Ig, immunoglobulin; NA, not applicable.

1 Significant heterogeneity (p<0.05).

**Table 5.** Pooled risk factors of WNV immunoglobulin M seropositivity in Nigeria

Variables (no. of studies)	No. of pooled subjects	No. of WNV–positive cases, n (%)	OR (95% CI)	p-value
Age (yr, n=4)				
≤17	61	14 (22.9)	3.73 (1.87, 7.45)	<0.001
>17	460	34 (7.4)	1.00 (reference)	
Sex (n=3)				
Male	154	16 (10.4)	1.32(0.68, 2.61)	0.411
Female	274	22 (8.0)	1.00 (reference)	
Residence (n=4)				
Suburban	429	43 (17.0)	1.35 (0.16, 3.11)	0.477
Urban	92	7 (7.6)	1.00 (reference)	
Level of education (n=2)				
No formal	73	7 (9.6)	4.31 (1.08, 17.2)	0.039
Primary	46	7 (15.2)	7.29 (1.80, 29.6)	0.005
High school	84	5 (5.9)	2.57 (0.59, 11.07)	0.204
Tertiary	125	3 (2.4)	1.00 (reference)	
Marital status (n=2)				
Single	146	13 (8.9)	1.95 (0.81, 4.71)	0.135
Married	189	9 (4.8)	1.00 (reference)	
Vegetation (n=7)				
Sahel	1,173	84 (7.2)	0.91 (0.59, 1.41)	0.673
Savannah	335	22 (6.6)	0.83 (0.47, 1.47)	0.522
Rainforest	371	29 (7.8)	1.00 (reference)	
Predominant temperature (°C, n=7)				
High (>38)	1,308	91 (6.9)	0.89 (0.61, 1.30)	0.563
Low (<25)	571	44 (7.7)	1.00 (reference)	
Use of insecticides (n=2)				
Yes	115	7 (6.1)	0.88 (0.35, 2.24)	0.798
No	220	15 (6.8)	1.00 (reference)	
Annual rainfall (mo, n=7)				
≥6	571	44 (7.7)	1.12 (0.77, 1.62)	0.563
<6	1,308	91 (6.9)	1.00 (reference)	

WNV, West Nile virus; OR, odds ratio; CI, confidence interval.
